# Ethnic differences in adverse iron status in early pregnancy: a cross-sectional population-based study

**DOI:** 10.1017/jns.2022.35

**Published:** 2022-06-01

**Authors:** Hugo G. Quezada-Pinedo, Florian Cassel, Martina U. Muckenthaler, Max Gassmann, Luis Huicho, Irwin K. Reiss, Liesbeth Duijts, Romy Gaillard, Marijn J. Vermeulen

**Affiliations:** 1 The Generation R Study Group, Erasmus University Medical Center, Rotterdam, The Netherlands; 2 Department of Pediatrics, Division of Neonatology, Erasmus MC, University Medical Center, Rotterdam, The Netherlands; 3 Department of Pediatrics, Erasmus MC, University Medical Center, Rotterdam, The Netherlands; 4 Department of Pediatric Hematology, Oncology and Immunology, University Hospital Heidelberg, Heidelberg, Germany; 5 Molecular Medicine Partnership Unit, University Hospital Heidelberg, Heidelberg, Germany; 6 Institute of Veterinary Physiology, Vetsuisse Faculty and Zurich Center for Integrative Human Physiology, University of Zurich, Zurich, Switzerland; 7 School of Medicine, Universidad Peruana Cayetano Heredia, Lima, Peru; 8 Centro de Investigación en Salud Materna e Infantil, Centro de Investigación para el Desarrollo Integral y Sostenible, Universidad Peruana Cayetano Heredia, Lima, Peru; 9 Department of Pediatrics, Division of Respiratory Medicine and Allergology, Erasmus MC, University Medical Center, Rotterdam, The Netherlands

**Keywords:** Ferritin, Haemoglobin, Iron-deficiency anaemia, Iron overload

## Abstract

We studied ethnic differences in terms of iron status during pregnancy between Dutch women and other ethnicities and explore to what extent these differences can be explained by environmental factors. This cross-sectional population-based study (2002–2006) was embedded in the Generation R study and included a total of 4737 pregnant women from seven ethnic groups (Dutch, Turkish, Moroccan, Cape Verdean, Surinamese-Hindustani, Surinamese-Creole and Antillean). Ethnicity was defined according to the Dutch classification of ethnic background. Ferritin, iron and transferrin were measured in early pregnancy. The overall prevalence of iron deficiency was 7 %, ranging from 4 % in both Dutch and Surinamese-Creoles, to 18 % in Turkish, Moroccan and Surinamese-Hindustani women. Iron overload was most prevalent in Surinamese-Creole (11 %) and Dutch (9 %) women. Socioeconomic factors accounted for 5–36 % of the differences. Income was the strongest socioeconomic factor in the Cape Verdean and Surinamese-Hindustani groups and parity for the Turkish and Moroccan groups. Lifestyle determinants accounted for 8–14 % of the differences. In all groups, the strongest lifestyle factor was folic acid use, being associated with higher iron status. In conclusion, in our population, both iron deficiency and iron overload were common in early pregnancy. Our data suggest that ethnic differences in terms of socioeconomic and lifestyle factors only partly drive the large ethnic differences in iron status. Our data support the development of more specific prevention programmes based on further exploration of socioeconomic inequities, modifiable risk and genetic factors in specific ethnic subgroups, as well as the need for individual screening of iron status before supplementation.

## Introduction

The World Health Organization (WHO) estimated that over 40 % of pregnant women worldwide are anaemic and declared that this a serious global public health problem^(1,2)^. Malnutrition, infection and genetic haemoglobinopathies are the main causes of anaemia, with half of the burden being related to iron deficiency^(3)^. Iron deficiency and anaemia during pregnancy are associated with adverse maternal and pregnancy outcomes^(4)^. For the unborn child, it increases the risk of growth restriction, premature birth and later cognitive dysfunction^(4,5)^.

To reach the Sustainable Development Goals (SDGs), one of the World Health Assembly Global Nutrition Targets for 2025 is a 50 % reduction of anaemia in women of reproductive age^(1,2,6)^. Despite the WHO recommendation to give iron supplementation to all pregnant women, global progress for reaching this target is not yet on track^(7)^. Not only in developing countries, but also in Europe, the prevalence of anaemia in pregnant women in showed an increasing trend in recent years^(8)^. With ongoing immigration and an increase in health inequities during the COVID-19 pandemic^(9)^, iron deficiency will remain a public health issue, which underlines the importance to find strategies to improve the efficiency of prevention programmes. The dissatisfying effects of many iron supplementation programmes may be due to adherence problems, but also to population-specific differences in the complex aetiology of iron deficiency.

The prevalence of iron deficiency varies geographically, with women in lower-income countries generally being at highest risk. Within continents and countries, maternal iron status varies between ethnic groups. For example, in the United States and Canada, Afro-American, Asian and Hispanic women were at higher risk of iron deficiency than non-Hispanic white women^(10)^. In some high-income populations, concern is not only focused on iron deficiency, but also on high iron stores, especially in iron-replete women receiving iron supplementation. Iron overload is associated with oxidative stress, pregnancy complications and impaired fetal growth^(11,12)^.

Little is known on the factors underlying the ethnic differences in iron status during pregnancy, which may not only be found in dietary iron intake and iron supplementation. Globally, screening and prevention programmes would benefit from a more comprehensive understanding of the relative contribution of socioeconomic, lifestyle and genetic factors that contribute to ethnic differences in adverse maternal iron status during pregnancy^(13,14)^.

The population-based prospective Generation R Study includes pregnant women from seven large ethnic groups living in the city of Rotterdam, The Netherlands^(15)^. The cohort offers a unique opportunity for studying ethnic differences in a setting comprising free antenatal health care, wide availability of iron-rich food and very low infection rates^(15)^. Thus, we aimed to study ethnic differences in terms of iron status during pregnancy, risk of iron deficiency and overload. Furthermore, we also aimed to explore to what extent these can be explained by environmental factors. We hypothesised that women with a migration background are at higher risk and that ethnic differences in iron status are largely explained by socioeconomic and lifestyle factors.

## Methods

### Study design

This cross-sectional study was embedded in the Generation R Study, a population-based prospective cohort study starting from fetal life onwards based in Rotterdam, The Netherlands^(15)^. Between April 2002 and January 2006, all pregnant women in Rotterdam were invited to participate in the Generation R Study, as described in detail previously^(15)^. Translated information packages and questionnaires were available for recruitment of different ethnicities. Of the 9778 participants in Generation R, we excluded women who gave no consent (*n* 802), with multiple participation (only first included, *n* 97), with no data available on ethnic background (*n* 501), belonging to small ethnical subgroups (*n* 1704), and without iron data (*n* 1937), leaving a total of 4737 subjects for the current analysis belonging to seven of the largest ethnic groups in The Netherlands (Supplementary Figure S1)^(16)^.

### Ethics

This study was conducted according to the guidelines laid down in the Declaration of Helsinki and all procedures involving human subjects were approved by the Medical Ethical Committee of the Erasmus MC, University Medical Centre in Rotterdam (MEC-2012-165-NL40020.078.12). Written informed consent was obtained from all women.

### Ethnic background

The Dutch classification of ethnic background was used, which was based on the country of birth of the participant as well as the participant's parents’ country of origin, as reported in the questionnaire completed at enrolment^(15)^. If the participant was born outside the Netherlands, her country of birth determined the ethnic background^(15)^. If she was born in the Netherlands, but one of her parents was born abroad, the last country of birth determined the ethnic background. If both her parents were born abroad, her mother's country of birth was leading. All others were categorised as Dutch. In this report, the term ‘migration background’ refers to not matching the definition of Dutch, meaning that migration, if any, was not within the last two generations.

### Iron status

Iron biomarkers were measured in non-fasting blood serum and plasma samples collected during early pregnancy and processed as described previously^(17)^. Gestational age at iron blood sampling was at mean 13⋅4 (sd 2⋅0) weeks, based on ultrasound examinations in early pregnancy^(15)^.

The primary analyses were focused on serum ferritin, which reflects intracellular stored iron and is the most widely used marker for iron deficiency and overload^(18)^. Serum ferritin was measured by an electrochemiluminescence immunoassay (ECLIA) on the Cobas e411 analyser (Roche, Almere, The Netherlands). Based on the most commonly applied definitions during pregnancy, iron deficiency was defined as ferritin <15 μg/l and iron overload as ferritin >150 μg/l^(3,18,19)^. Ferritin is a positive acute-phase protein, that is elevated in the presence of infection or inflammation, regardless of iron status^(20,21)^. As this may occur in case of infection, but also in low-grade inflammation associated with adiposity, we included C-reactive protein (CRP) as a measure of inflammation^(22)^.

To allow comparison with studies that used other measures of iron status, secondary analyses were done on transferrin saturation and haemoglobin concentration. Transferrin saturation, reflecting the iron-bound part of the total iron-binding capacity, was calculated using the formula (serum iron×100)/transferrin×25⋅1^(17)^. Serum iron was determined by colorimetric assay and transferrin by immunoturbidimetric assay, both measured with C502 on the Cobas 8000 (Roche, Almere, The Netherlands). Haemoglobin, as well as high sensitivity CRP concentrations, were measured in venous EDTA plasma samples as previously described^(17,23)^. Anaemia was defined as haemoglobin <11 g/dl, and iron-deficiency anaemia as ferritin <15 μg/l + haemoglobin <11 g/dl^(3)^. To capture the full range of inflammation, and meaningful cut-offs are not agreed upon, we used CRP as a continuous variable^(24,25)^.

### Socioeconomic and lifestyle factors

A conceptual framework for ethnical differences in iron status during early pregnancy, based on the literature, is shown in Supplementary Figure S2. The present study focuses on the environmental (socioeconomic and lifestyle) factors, as the impact of known genetic factors on the prevalence of iron deficiency has been reported to be low^(10)^. Information was taken from questionnaires during pregnancy at enrolment. The socioeconomic factors included total household income (<1200 €/month, i.e. below the Dutch social security level in 2002–6; 1200–2200 €/month or >2200 €/month, i.e. above the approximate monthly general labour income in 2002–6)^(26,27)^, educational level (low: no education, primary education; middle: secondary phase 1 or 2 finished; and high: higher vocational training or university degree) and parity (nulliparity yes/no). The lifestyle-related factors included dietary iron intake (mg/d), based on a validated semi-quantitative food frequency questionnaire, recalling consumption of 293 foods in the past 3 months^(28)^. Food intakes (g/d) were based on frequency and estimated portion sizes, using food photographs, Dutch household measures, methods of preparations and additions^(28)^. Iron supplement use (based on reported multivitamin use) (yes/no), folic acid supplement use (no/periconceptional use/in first 10 weeks of pregnancy), pre-pregnancy body mass index (BMI), smoking (yes/no) and psychological distress (yes/no)^(29)^. The latter was added based on animal and human studies suggesting that maternal stress may influence iron absorption^(30)^ and was defined as a score >0⋅71 on the overall psychological distress scale using the Brief Symptom Inventory, as previously described^(31)^.

### Statistical analysis

In a non-response analysis, the characteristics of women with missing data on ethnicity or iron status (*n* 4142 excluded) were compared with women participating in the study (*n* 4737 included) (Supplementary Table S1). Descriptive statistics were determined for the total study population, as well as per ethnic group, as means with standard deviation (sd) or median (interquartile range) for normally and non-normally distributed variables, respectively, and absolute numbers (*n*) with percentages (%) for categorical variables. Characteristics were compared using *T* test, Mann–Whitney *U* test and *χ*^2^ tests as appropriate and overall comparison between all groups using ANOVA, Kruskal–Wallis and *χ*^2^ test.

For the primary analyses, linear and logistic regression models were built to assess the association of ethnic background with serum ferritin, iron deficiency and iron overload. To meet the homoscedasticity assumption of linear regression, ferritin was natural log-transformed. To enable comparison of the effect estimates, standard deviation scores (SDS) (value − mean)/sd) for the iron biomarkers were calculated. In the basic model (model 1), we adjusted for the potential confounders of age, gestational age at iron blood sampling and CRP. Iron status is reported to be lower at younger age, and it decreases with gestational age due to haemodilution and iron mobilisation during pregnancy^(20)^. CRP was added to account for the effect of inflammation on increasing ferritin levels^(20)^. To determine the following models, in model 1, we selected potential explanatory factors based on the literature that led to a substantial change in the effect estimates (i.e. ≥10 % change). In the socioeconomic model (model 2), we adjusted model 1 additionally for three socioeconomic factors: monthly total household income, education and parity. In the lifestyle model (model 3), we adjusted model 1 additionally for the lifestyle factors dietary iron intake, iron supplement use, folic acid supplement use, BMI, smoking and psychological distress. In the full model (model 4), we adjusted model 1 additionally for both the socioeconomic and lifestyle factors. Fetal sex did not change the basic model and was therefore not included in any of the models. Similarly, we assessed the association of ethnic background and the secondary outcomes (transferrin saturation, haemoglobin, anaemia and iron-deficiency anaemia) in four different models. Effect estimates were expressed in betas (*β*, reflecting change in ferritin SDS) or odds ratios (ORs, reflecting the odds for a binary outcome) with 95 % Confidence Interval (95 % CI). In the ethnic groups with significantly different ferritin in the full model, we explored the relative contribution of the socioeconomic and lifestyle determinants, using the Oaxaca-Blinder analysis (Oaxaca package in R) and ranked the factors^(32)^.

Variables were complete for >79 % each. Missing covariates showed no clear missing pattern and were replaced by multiple imputation (*m* 10) using the Fully Conditional Specification method (MICE package in R)^(33)^. Given the hypothesis-driven analysis and the strong correlation between outcomes, no adjustment for multiple testing was applied^(34)^. All statistical analyses were conducted using R version 4.0.2 (R Foundation, Vienna, Austria). *P*-values <0⋅05 (two-tailed) were considered statistical significant.

## Results

### Descriptive data

The characteristics of the total study population and of ethnic subgroups are shown in Table 1. The mean age of the participants ranged between 26⋅8 years in the Turkish as well as the Antillean groups, to 31⋅2 years in the Dutch group. Over half of the participants were nulliparous (57⋅4 %). Median CRP levels were 4⋅6 (IQR 2⋅5, 8⋅2). Ethnical differences were seen in the prevalence of iron deficiency and overload, as well as in household income, educational levels and lifestyle factors, with the Dutch subgroup generally having the most favourable characteristics.

**Table 1.** Population characteristics for total study population and by ethnic subgroup

	Total (*n* 4737)	Dutch (*n* 3112)	Turkish (*n* 474)	Moroccan (*n* 352)	Cape Verdean (*n* 244)	Surinamese-Hindustani (*n* 212)	Surinamese-Creole (*n* 170)	Antillean (*n* 173)
*Basic*								
Age (years)	29⋅9 (5⋅0)	31⋅2 (4⋅3)	26⋅8 (4⋅7)**	28⋅0 (5⋅3)**	27⋅3 (5⋅9)**	27⋅3 (4⋅8)**	28⋅1 (6⋅2)**	26⋅8 (5⋅3)**
GA at blood sampling (weeks)	13⋅4 (2⋅0)	13⋅2 (1⋅9)	13⋅8 (2⋅1)**	14⋅2 (2⋅2)**	14⋅0 (1⋅9)**	13⋅7 (2⋅1)*	**13⋅5 (2⋅1)**	13⋅6 (2⋅3)*
CRP (mg/l)	4⋅6 (2⋅5, 8⋅2)	4⋅1 (2⋅3, 7⋅4)	5⋅3 (2⋅9, 8⋅8)	6⋅3 (3⋅4, 10⋅1)*	4⋅8 (3⋅0, 8⋅9)	6⋅5 (3⋅6, 11⋅5)*	5⋅4 (2⋅6, 9⋅8)	4⋅8 (2⋅5, 9⋅4)
*Socioeconomic status*								
Monthly household income, *n* (%)			***P* < 0⋅001**	***P* < 0⋅001**	***P* < 0⋅001**	***P* < 0⋅001**	***P* < 0⋅001**	***P* < 0⋅001**
<1200 €	668 (17⋅3)	148 (5⋅4)	145 (41⋅8)	107 (51⋅2)	95 (59⋅0)	51 (34⋅5)	54 (46⋅6)	68 (54⋅0)
1200–2200 €	954 (24⋅7)	565 (20⋅5)	144 (41⋅5)	82 (39⋅2)	50 (31⋅1)	51 (34⋅5)	30 (25⋅9)	32 (25⋅4)
>2200 €	2244 (58⋅0)	2046 (74⋅2)	58 (16⋅7)	20 (9⋅6)	16 (9⋅9)	46 (31⋅1)	32 (27⋅6)	26 (20⋅6)
Educational level, *n* (%)			***P* < 0⋅001**	***P* < 0⋅001**	***P* < 0⋅001**	***P* < 0⋅001**	***P* < 0⋅001**	***P* < 0⋅001**
Low	446 (9⋅7)	109 (3⋅5)	135 (30⋅8)	91 (28⋅7)	50 (22⋅5)	21 (10⋅2)	12 (7⋅5)	28 (17⋅0)
Intermediate	2108 (46⋅0)	1150 (37⋅4)	236 (53⋅8)	185 (58⋅4)	151 (68⋅0)	156 (75⋅7)	123 (76⋅4)	107 (64⋅8)
High	2028 (44⋅3)	1813 (59⋅0)	68 (15⋅5)	41 (12⋅9)	21 (9⋅5)	29 (14⋅1)	26 (16⋅1)	30 (18⋅2)
Nulliparous (%)	2710 (57⋅4)	1887 (60⋅8)	237 (50⋅1)**	136 (39⋅0)**	136 (56⋅4)	130 (61⋅6)	**87 (51⋅2)**	97 (57⋅1)
*Lifestyle factors*								
Dietary iron intake (mg/d)	11⋅1 (8⋅7, 13⋅5)	11⋅8 (9⋅6, 14⋅1)	8⋅9 (7⋅2, 11⋅4)**	9⋅1 (7⋅2, 11⋅7)**	8⋅7 (6⋅7, 10⋅8)**	8⋅8 (7⋅1, 11⋅9)**	9⋅6 (7⋅5, 12⋅1)**	8⋅9 (6⋅7, 10⋅7)**
Iron supplement use, yes (%)	1201 (29⋅2)	980 (35⋅8)	55 (14⋅1)**	42 (14⋅2)**	32 (15⋅8)**	26 (14⋅1)**	29 (19⋅6)**	37 (24⋅5)*
Folic acid supplement use, *n* (%)			***P* < 0⋅001**	***P* < 0⋅001**	***P* < 0⋅001**	***P* < 0⋅001**	***P* < 0⋅001**	***P* < 0⋅001**
No use	918 (24⋅3)	266 (10⋅4)	193 (52⋅3)	173 (65⋅8)	110 (61⋅1)	70 (42⋅7)	50 (38⋅8)	56 (44⋅1)
Periconceptional use	1673 (44⋅3)	1442 (56⋅6)	75 (20⋅3)	40 (15⋅2)	19 (10⋅6)	37 (22⋅6)	24 (18⋅6)	36 (28⋅3)
First 10 weeks use	1188 (31⋅4)	839 (32⋅9)	101 (27⋅4)	50 (19⋅0)	51 (28⋅3)	57 (34⋅8)	55 (42⋅6)	35 (27⋅6)
BMI (kg/m)	24⋅6 (4⋅5)	24⋅1 (4⋅1)	25⋅7 (5⋅0)**	26⋅0 (4⋅5)**	25⋅0 (4⋅5)*	24⋅2 (5⋅2)	25⋅7 (5⋅4)**	26⋅1 (5⋅6)**
Smoking during pregnancy, yes (%)	1228 (28⋅3)	776 (27⋅0)	193 (44⋅2)**	21 (6⋅8)**	76 (36⋅9)*	52 (25⋅7)	59 (37⋅1)*	51 (32⋅1)
Psychological distress, yes (%)	357 (9⋅2)	100 (3⋅7)	99 (29⋅7)**	48 (19⋅9)**	47 (28⋅0)**	23 (13⋅9)**	18 (14⋅5)**	22 (16⋅5)**

Values are means (sd), medians (25th, 75th percentile) or absolute numbers (percentages) based on observed data. BMI, Pre-pregnancy body max index; CRP, C-reactive protein; GA, gestational age. Values that are significantly different as compared to the Dutch reference group are indicated in bold (*P* < 0⋅05), with * (*P* < 0⋅01) or ** (*P* < 0⋅001), based on *T* test, Mann–Whitney *U* test and *χ*^2^ test.

In the total study population, iron deficiency and overload were prevalent in 330 (7⋅0 %) and 318 (6⋅7 %) women, respectively (Table 2). Dietary iron intake was lower in the iron-deficient group as compared to the normal group (median 10⋅3 *v*. 11⋅2 mg/d, respectively, *P* = 0⋅001), but not significantly higher in the iron overload group (median 11⋅0, *P* = 0⋅06) (Supplementary Table S2). Iron supplementation was more common in women with iron overload (34 %) than in those with iron deficiency (15 %) (*P* < 0⋅001). The prevalence of iron deficiency was highest (around 18 %) in the Turkish, Moroccan and Surinamese-Hindustani groups. In the Cape Verdean and Antillean groups, the prevalence of iron deficiency was 7⋅8 and 5⋅8 %, respectively. In the Dutch and the Surinamese-Creoles, the prevalence of iron deficiency was lowest (3⋅5 % and 4⋅1 %, respectively) and iron overload highest (8⋅7 % and 10⋅6 %, respectively). The distribution of serum ferritin by ethnic group is shown graphically in Supplementary Figure S3.

**Table 2.** Ferritin concentrations and the prevalence of iron deficiency and overload during early pregnancy by ethnic background

		All	Dutch	Turkish	Moroccan	Cape Verdean	Surinamese- Hindustani	Surinamese- Creole	Antillean
	*N*	4737	3112	474	352	244	212	170	173
Serum ferritin, μg/l	median	52⋅9	60⋅9	**33⋅2**	**32⋅4**	**45⋅3**	**31⋅8**	59⋅8	**52⋅6**
	(p25,p75)	(30⋅6, 85⋅1)	(37⋅5, 95⋅0)	(17⋅7, 57⋅6)**	(18⋅4, 55⋅3)**	(26⋅0, 73⋅3)**	(17⋅7, 53⋅1)**	(31⋅7, 96⋅9)	**(29⋅3, 93⋅9)**
Iron deficiency^a^	*n* (%)	330 (7⋅0)	110 (3⋅5)	83 (17⋅5)**	63 (17⋅9)**	**19 (7⋅8)**	38 (17⋅9)**	7 (4⋅1)	10 (5⋅8)
Iron overload^b^	*n* (%)	318 (6⋅7)	270 (8⋅7)	5 (1⋅1)**	3 (0⋅9)**	8 (3⋅3)*	3 (1⋅4)*	18 (10⋅6)	11 (6⋅4)

*N*, number of participants.

a Defined as serum ferritin <15 μg/l; normal serum ferritin (15–150 μg/l).

b Defined as serum ferritin >150 μg/l.

Values that are significantly different as compared to the Dutch reference group are indicated in bold (*P* < 0⋅05), with * (*P* < 0⋅01) or ** (*P* < 0⋅001), based on *T* test, Mann–Whitney *U* test and *χ*^2^ test.

### Multivariable analyses

As compared to the Dutch reference group, most ethnic backgrounds were associated with increased odds for iron deficiency after adjusting for age, gestational age at blood sampling and CRP (Table 3). This increase was 5-fold in Turkish OR 5⋅1 [95 % CI 3⋅7, 7⋅1]), Moroccan (OR 5⋅2 [3⋅7, 7⋅3]) and Surinamese-Hindustani (OR 5⋅5 [3⋅6, 8⋅3]), which attenuated but remained highly significant after adjustment for socioeconomic and lifestyle factors (OR 3⋅6 [2⋅4, 5⋅3], 3⋅1 [2⋅0, 4⋅8] and 4⋅4 [2⋅8, 7⋅0], respectively). Cape Verdean background was associated with a 2-fold increase in odds for iron deficiency OR (95 % CI) 1⋅5 (0⋅8, 2⋅6), which was no longer significant after adjustment for the environmental factors. The odds for iron overload were highest in the Dutch reference group as well as the Surinamese-Creole group OR (95 % CI) 1⋅8 (1⋅0, 3⋅2).

**Table 3.** Multivariable analyses of the association between ethnicity and ferritin, iron deficiency, and iron overload during early pregnancy

Total (*n* 4737)	Dutch (*n* 3112)	Turkish (*n* 474)	Moroccan (*n* 352)	Cape Verdean (*n* 244)	Surinamese-Hindustani (*n* 212)	Surinamese-Creole (*n* 170)	Antillean (*n* 173)
Serum ferritin (SDS)							
		*β* (95 % CI)	*β* (95 % CI)	*β* (95 % CI)	*β* (95 % CI)	*β* (95 % CI)	*β* (95 % CI)
Basic model	Ref	−0⋅7 (−0⋅8, −0⋅6)**	−0⋅7 (−0⋅8, −0⋅6)**	−0⋅3 (−0⋅4, −0⋅2)**	−0⋅8 (−0⋅9, −0⋅7)**	0⋅0 (−0⋅2, 0⋅1)	**−0⋅1 (−0⋅3, −0⋅0)**
se model	Ref	−0⋅5 (−0⋅6, −0⋅4)**	−0⋅5 (−0⋅6, −0⋅4)**	**−0⋅1 (−0⋅3, −0⋅0)**	−0⋅7 (−0⋅8, −0⋅6)**	0⋅1 (−0⋅0, 0⋅3)	0⋅0 (−0⋅1, 0⋅2)
LS model	Ref	−0⋅7 (−0⋅8, −0⋅6)**	−0⋅7 (−0⋅8, −0⋅5)**	−0⋅2 (−0⋅4, −0⋅1)**	−0⋅7 (−0⋅9, −0⋅6)**	0⋅0 (−0⋅2, 0⋅1)	−0⋅1 (−0⋅3, 0⋅0)
Full model	Ref	−0⋅6 (−0⋅7, −0⋅5)**	−0⋅5 (−0⋅6, −0⋅4)**	**−0⋅1 (−0⋅3, −0⋅0)**	−0⋅7 (−0⋅8, −0⋅5)**	0⋅1 (−0⋅0, 0⋅3)	0⋅0 (−0⋅1, 0⋅2)
Iron deficiency							
		OR (95 % CI)	OR (95 % CI)	OR (95 % CI)	OR (95 % CI)	OR (95 % CI)	OR (95 % CI)
Basic model	1⋅0	5⋅1 (3⋅7, 7⋅1)**	5⋅2 (3⋅7, 7⋅3)**	2⋅0 (1⋅2, 3⋅4)*	5⋅5 (3⋅6, 8⋅3)**	1⋅1 (0⋅5, 2⋅4)	1⋅5 (0⋅8, 2⋅9)
se model	1⋅0	3⋅8 (2⋅6, 5⋅5)**	3⋅7 (2⋅5, 5⋅5)**	1⋅6 (0⋅9, 2⋅8)	4⋅9 (3⋅2, 7⋅7)**	0⋅9 (0⋅4, 2⋅0)	1⋅2 (0⋅6, 2⋅4)
LS model	1⋅0	4⋅3 (3⋅0, 6⋅2)**	3⋅8 (2⋅6, 5⋅7)**	1⋅6 (0⋅9, 2⋅8)	4⋅6 (3⋅0, 7⋅1)**	0⋅9 (0⋅4, 2⋅1)	1⋅3 (0⋅6, 2⋅6)
Full model	1⋅0	3⋅6 (2⋅4, 5⋅3)**	3⋅1 (2⋅0, 4⋅8)**	1⋅5 (0⋅8, 2⋅6)	4⋅4 (2⋅8, 7⋅0)**	0⋅8 (0⋅4, 1⋅9)	1⋅1 (0⋅5, 2⋅3)
Iron overload							
		OR (95 % CI)	OR (95 % CI)	OR (95 % CI)	OR (95 % CI)	OR (95 % CI)	OR (95 % CI)
Basic model	1⋅0	0⋅1 (0⋅1, 0⋅3)**	0⋅1 (0⋅0, 0⋅3)**	**0⋅4 (0⋅2, 0⋅9)**	0⋅2 (0⋅1, 0⋅5)*	1⋅4 (0⋅8, 2⋅3)	0⋅8 (0⋅4, 1⋅5)
se model	1⋅0	0⋅2 (0⋅1, 0⋅5)**	0⋅2 (0⋅1, 0⋅6)*	0⋅6 (0⋅3, 1⋅3)	0⋅2 (0⋅1, 0⋅6)*	2⋅1 (1⋅2, 3⋅6)*	1⋅1 (0⋅6, 2⋅2)
LS model	1⋅0	0⋅1 (0⋅1, 0⋅3)**	0⋅1 (0⋅0, 0⋅3)**	**0⋅5 (0⋅2, 1⋅0)**	0⋅2 (0⋅1, 0⋅5)*	1⋅3 (0⋅8, 2⋅2)	0⋅8 (0⋅4, 1⋅5)
Full model	1⋅0	0⋅2 (0⋅1, 0⋅4)**	0⋅2 (0⋅0, 0⋅5)*	0⋅6 (0⋅3, 1⋅3)	0⋅2 (0⋅1, 0⋅6)*	**1⋅8 (1⋅0, 3⋅2)**	1⋅0 (0⋅5, 2⋅0)

Values indicate betas (*β*, reflecting change in ferritin SDS) or odds ratios (ORs) with 95 % confidence interval (95 % CI), derived from linear (for serum ferritin SDS as a continuous variable) and logistic regression models (for iron deficiency and iron overload as categorical outcomes) based on multiple imputed data, reported per ethnic group compared with the Dutch (reference) group. Normal serum ferritin (15–150 μg/l). Iron deficiency is defined as serum ferritin <15 μg/l (yes/no), and iron overload as serum ferritin >150 μg/l (yes/no). The basic model was adjusted for age, gestational age at iron blood sampling and C-reactive protein. The socioeconomic (se) model was adjusted for the factors in the basic model and for income, education and parity. The lifestyle (LS) model was adjusted for the factors in the basic model and dietary iron intake, iron supplement use, folic acid supplement use, body mass index, smoking during pregnancy and psychological distress. The full model included all these determinants named (see Supplementary Figure S2). Values that are significant are indicated in bold (*P* < 0⋅05), with * (*P* < 0⋅01) or ** (*P* < 0⋅001).

**Fig. 1.**
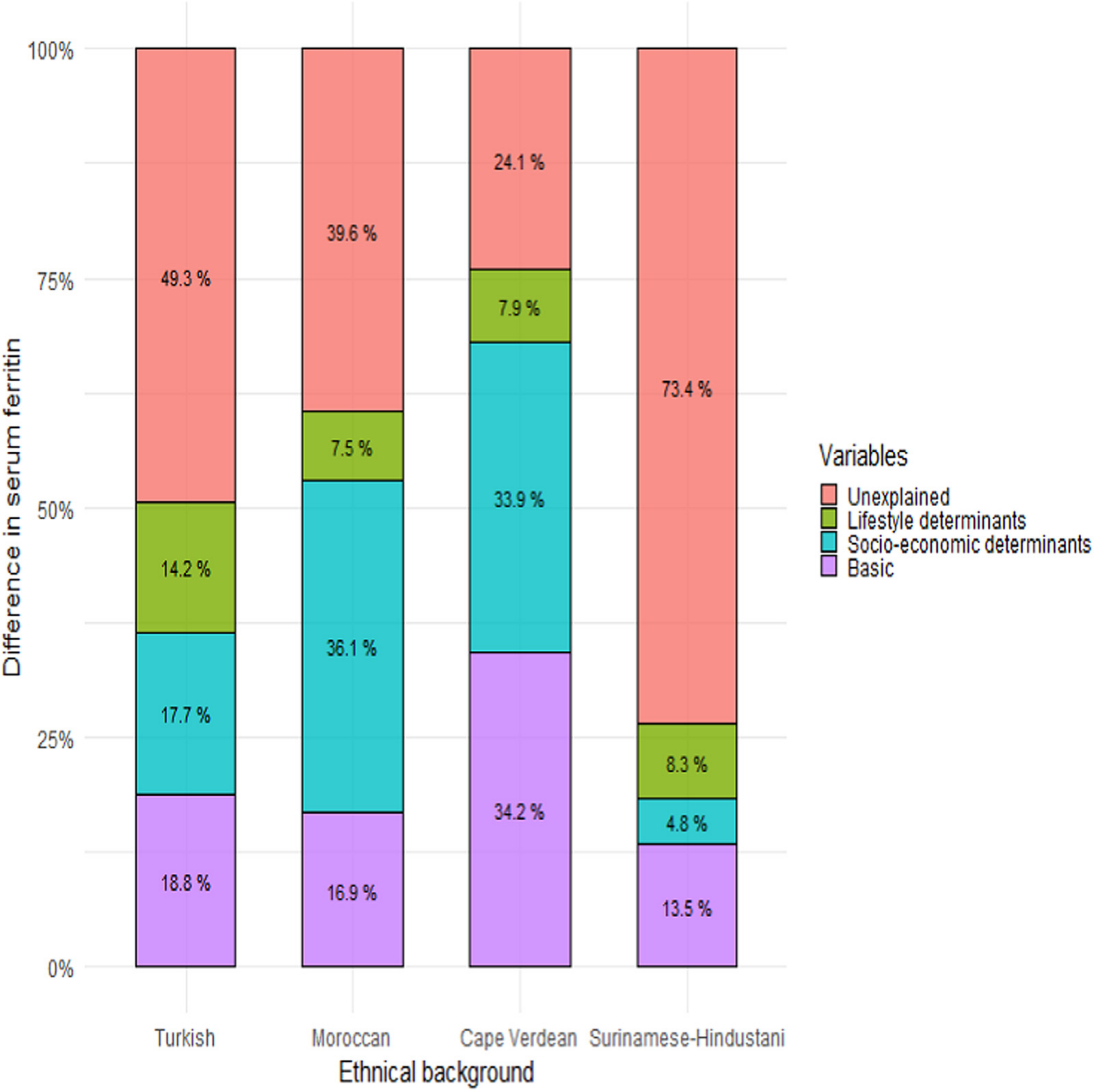
Oaxaca-Blinder decomposition explaining differences in early pregnancy. Relative contributions of determinants to the lower mean maternal serum ferritin concentration in early pregnancy as compared to the Dutch reference group. Basic determinants: age, gestational age at iron blood sampling, C-reactive protein; socioeconomic determinants: monthly household income, education, parity; lifestyle determinants: dietary iron intake, iron supplementation, folic acid supplement use, pre-pregnancy body mass index, smoking during pregnancy, psychological distress (see also Supplementary Table S3).

The differences in serum ferritin were further explored in the Turkish, Moroccan, Cape Verdean and Surinam-Hindustani groups, as compared to the Dutch reference group. The relative contribution of factors varied by the group (Fig. 1 and Supplementary Table S3). The basic factors, including age, gestational age and CRP, accounted for 14–34 % of the differences. On top of these basic determinants, socioeconomic factors (income, parity and education), accounted for 5–36 % of the differences in serum ferritin. Among the three socioeconomic factors, income was the strongest associated factor for the Cape Verdean and Surinamese-Hindustani groups and parity for the Turkish and Moroccan groups. The lifestyle determinants (diet, supplementation, folic acid use, BMI, smoking and psychological distress) accounted for 8–14 % of the differences. In all groups, of all the lifestyle factors associated with serum ferritin differences, the strongest factor was folic acid use. The order of the other lifestyle factors differed by group, with iron supplementation and psychological distress being among the top 4 strongest lifestyle factors in all four groups. The combination of all factors did not fully explain the ethnic differences in ferritin. In the Cape Verdean, the combination of factors explained 76 % of the difference, leaving 24 % unexplained. In the other groups even larger parts remained unexplained, being most extreme in the Surinamese-Hindustani women in whom 73 % of the difference in ferritin remained unexplained.

### Secondary analyses

Transferrin saturation and haemoglobin levels were lower in all groups, as compared to the Dutch reference while the prevalence of anaemia varied between groups, ranging from 4 % (Dutch) to 28 % (Surinamese-Creole) (Supplementary Table S4). The prevalence of iron-deficiency anaemia ranged between 0⋅4 % (Dutch) and 8⋅3 % (Surinamese-Hindustani). Adjustment for socioeconomic or lifestyle factors had no or little effect on the association between ethnicity and transferrin saturation or haemoglobin as continuous measures (Supplementary Table S5). The association of ethnic background (OR 2⋅0 (1⋅2, 3⋅4)) and anaemia was strongest in the Surinamese-Creole group (OR 9⋅2 [6⋅1, 14⋅0]), but also strong in the other groups, with ORs ranging from 2⋅3 to 5⋅4 (Supplementary Table S6). The models showed no substantial effect of socioeconomic and lifestyle factors on the associations with anaemia, which was therefore not further explored.

Ethnicity was also associated with iron-deficiency anaemia, which was partly explained by the environmental factors (Supplementary Tables S4 and S6). For example, the high odds for iron-deficiency anaemia in the Surinamese-Hindustani group (OR 17⋅0 [7⋅4, 38⋅9]) decreased, but remained high, after adjustment for the socioeconomic and lifestyle factors (OR 10⋅5 [4⋅1, 26⋅7]).

## Discussion

In our population-based prospective cohort, large differences between the seven ethnic subgroups were found in early pregnancy iron status. Iron deficiency was more common in women with a migration background than in the Dutch reference group. In the Dutch and Surinamese-Creole group, iron overload was relatively common. Ethnical differences in ferritin levels and in prevalence of iron deficiency and iron overload remained after adjustment for socioeconomic and lifestyle-related factors. These factors only partly explained the associations of ethnic background and adverse iron status.

### Comparison with the literature

To our knowledge, the current findings represent the largest European cohort study exploring the association of ethnicity and adverse iron status during pregnancy^(35)^. The 7 % overall prevalence of iron deficiency in early pregnancy is similar to a report from Belgium (6 %), but lower than in other European studies (19 % in Germany and Switzerland, 33 % in Norway). This may reflect differences in populations and health care, but may also be explained by our measurements taken earlier during pregnancy or stricter definitions^(36–39)^.

Our study confirms findings from previous North American studies, showing that ethnic minority background is an important risk factor for iron deficiency^(10,40,41)^. The HEIRS Study, that included over 60 000 pregnant and non-pregnant women, showed that the risk of iron deficiency was higher in Hispanics and black, as compared to white and Asian women^(10)^. European studies consistently showed that pregnant women with a non-Western migration background had higher risk of iron deficiency than women with a Western background^(36–38)^. Instead of grouping per continent or large regions, the present study adds information on different ethnic groups defined on country of birth, living in a large urban Western city, and exploring associated factors. Comparison by country of origin was only reported previously in a study from Singapore, showing higher risk in pregnant women from Malaysia and India than from China^(42)^. They also observed that, in addition to ethnicity, education, parity and iron supplementation were associated with iron deficiency.

### Interpretation of results

In the four ethnic groups with the highest prevalence of iron deficiency, we observed that socioeconomic and lifestyle factors explained differences in serum ferritin to various extents and that ranking of contributing factors varied per group. In each group compared, the three most important lifestyle factors included folic acid and iron supplementation, suggesting that there is room for improvement of supplementation policies in these subgroups.

Despite the large number and wide range of environmental factors being taken into account, a substantial part of the difference remained unexplained. A limitation of the present study is the lack of genetic data, which may partly account for this finding. Our anaemia data in the three subgroups with a predominantly shared African ancestry (Surinamese-Creole, Antillean and Cape Verdean) would support this idea. Their typical pattern with a high prevalence of anaemia with relatively normal iron status, suggests that hereditary haematological diseases, like sickle cell anaemia, may play a role^(41)^. The extremely high prevalence of anaemia in Surinamese-Hindustani women seems largely explained by iron deficiency. We can speculate that in the women with anaemia without iron deficiency, hereditary hemoglobinopathies, such as thalassaemia may be involved. Hemoglobinopathies are generally not associated with iron deficiency, but with normal or mildly increased of ferritin levels, or even highly increased levels in case of multiple erythrocyte transfusions. Data on the prevalence of hemoglobinopathies in our ethnical subgroups are lacking, but national neonatal screening programmes suggest an increase of the prevalence related to immigration from endemic regions^(43,44)^. Other genetic mechanisms underlying ethnic differences in iron status may be found in common genetic variants associated with iron metabolism^(45)^. For example, the variants in the hemochromatosis (HFE) gene lead to increased iron absorption and may be more prevalent in specific ethnic groups such as Caucasians^(46–48)^. However, the impact of these variants on the prevalence of iron deficiency seems limited^(10)^.

The prevalence of iron overload in our population was high (7 %) as compared to previous literature (0–3 %)^(49,50)^. To explain this, further study is needed in the groups at highest risk of overload, specifically to clarify the role of diet and supplementation in the Dutch and Surinamese-Creole women. Cautiously interpreting our observational data, our data support the idea that in specific groups screening of iron status is needed before starting iron supplementation or over-the-counter iron-enriched multivitamin preparations. The need for individual screening of iron status is further supported by our secondary analysis which showed that patterns of anaemia do not correlate well with iron deficiency, suggesting that testing for haemoglobin alone is not sufficient. To reduce the global public health problem of iron deficiency and anaemia, the WHO recommends iron supplementation for all pregnant women. The effectiveness and safety of this strategy may improve by specifically addressing subgroups at risk, such as those with a migration background. Our study not only supports further investigation of ethnical differences in preventable risk factors for iron deficiency but also the further exploration of the safety of iron supplementation in subgroups both at risk and not at risk of iron overload. The development of screening and supplementation recommendations based on individual iron status and risk profile may be helpful in improving maternal and neonatal outcome, especially in multi-ethnic populations.

### Strengths and limitations

Strengths of our study include the prospective design and the large population-based multi-ethnic study sample with availability of iron biomarkers in early pregnancy and many important socioeconomic and lifestyle factors. Women participating in the Generation R Study, and more specifically the 54 % included in the present study, were relatively highly educated and healthy. Although differences may seem small and of little clinical relevance, selective inclusion of those at lowest risk might have reduced power in the ethnic minority groups and may have diluted some of the effects.

Our data were collected over a decade ago and, with time, may become outdated due to ongoing immigration and emigration. However, national population data support that the ethnical groups studied still represent seven of the largest subpopulations in The Netherlands, year 2021^(51)^.

We used clear definitions based on country of birth of participants and their parents. Women of second or higher generation after migration were categorised as Dutch, in line with the assumption that after migration it takes only a few generations to adapt to the new environment. However, genetic differences can persist, and in relatively closed cultural subgroups adaptation may take longer. By including not only native Dutch but also women with a, albeit less recent, migration history in our reference group, we may have diluted the differences between the groups. Less clear were the definitions of iron deficiency and iron overload, for which no meaningful cut-offs are agreed on in pregnancy^(18,52)^. Although cut-offs are arbitrary, we applied the most common definitions in clinical reports of <15 and >150 mg/l for iron deficiency and iron overload, respectively. To take into account the gradual decrease in iron storage during pregnancy, we included gestational age at blood sampling in the models.

Despite the use of validated questionnaires for the socioeconomic and lifestyle factors, reporting and recall bias cannot be ruled out. The food frequency questionnaire used is only validated in non-pregnant white women, and did not take iron bio-availability and iron-fortified foods into account, which may have led to misclassification of dietary iron intake in the ethnic minority groups. Speculating on the potential impact on our results is difficult, because our dietary iron intake data are similar to previous European cohort studies^(53)^. Despite the adjustment for many important determinants of iron status, residual confounding attributed to unmeasured factors cannot be ruled out in this observational study, for example by unmeasured dietary habits and iron supplementation. Such supplementation may have been underestimated by using only self-reported multivitamin preparation as a proxy. Data on prescribed iron preparation was not available. We expect that the effect on our results is small, as prescriptions are usually not started before the first trimester and the guidelines gave no recommendation on iron supplements by ethnicity.

In this cross-sectional study, no data were available on iron status during later pregnancy, when the risk of iron deficiency is highest. Longitudinal data are of specific interest if access to health care and use of iron supplementation during pregnancy differ between ethnic groups. For translation into practice, further studies are needed to explore differences in effects and safety of screening, prevention and treatment in different ethnic groups and their impact on maternal and neonatal health.

## Conclusion

Iron deficiency and anaemia is a global issue, seriously affecting millions of pregnant women and their off-spring. The present study demonstrated ethnic differences in iron status during early pregnancy in a multi-ethnic general urban European population, with women of a non-Dutch ethnic background having a high risk of iron deficiency and low risk of iron overload. The ethnic differences in iron status were only partly explained by the socioeconomic and lifestyle determinants. This underlines the need for further exploration of the underlying mechanisms, including genetics and modifiable risk factors, to improve screening and prevention programmes for pregnant women at risk.

## Supplementary material

For supplementary material accompanying this paper visit https://doi.org/10.1017/jns.2022.35. click here to view supplementary material
