# Supplementary material for: Ethnic differences in adverse iron status in early pregnancy: a cross-sectional population-based study
Source: J Nutr Sci. 2022 Jun 1;11:e39. doi: 10.1017/jns.2022.35 (PMC9161035; doi:10.1017/jns.2022.35)
Supplement: Supplementary file 1 [file S2048679022000350sup001.docx]

**On-line Supplementary Material**

**Ethnic differences in adverse iron status in early pregnancy:**

**a cross-sectional population-based study**

**Short title:** Ethnicity and maternal iron status in pregnancy

Hugo G. Quezada-Pinedo^1,2^, Florian Cassel^3^, Martina U. Muckenthaler^4^; Max Gassmann^5,7^, Luis Huicho^6,7^, Irwin K. Reiss^1,3^, Liesbeth Duijts^2,8^_,_ Romy Gaillard^1,3^, Marijn J. Vermeulen^1,2,3^

^1^The Generation R Study Group; ^2^Department of Pediatrics, Division of Neonatology, Erasmus MC, University Medical Center, Rotterdam, The Netherlands; ^3^Department of Pediatrics, Erasmus MC, University Medical Center, Rotterdam, The Netherlands; ^4^Department of Pediatric Hematology, Oncology and Immunology, University Hospital Heidelberg, Germany; Molecular Medicine Partnership Unit; ^5^Institute of Veterinary Physiology, Vetsuisse Faculty and Zurich Center for Integrative Human Physiology, University of Zurich, Zurich, Switzerland; ^6^Centro de Investigación en Salud Materna e Infantil, Centro de Investigación para el Desarrollo Integral y Sostenible, Universidad Peruana Cayetano Heredia, Lima, Peru; ^7^School of Medicine, Universidad Peruana Cayetano Heredia, Lima, Peru; ^8^Department of Pediatrics, Division of Respiratory Medicine and Allergology, Erasmus MC, University Medical Center, Rotterdam, The Netherlands.

**Supplementary Table 1.** Comparison of characteristics between non-participants and participants in the analysis.

|  | Non-participants  (n= 4142) | Participants  (n= 4737) | p-value |
| --- | --- | --- | --- |
| Ethnicity |  |  |  |
| Dutch | 984 (27.0) | 3112 (65.7) | <0.001 |
| Other western | 709 (19.5) | 0 (0.0) |  |
| Turkish | 298 (8.2) | 474 (10.0) |  |
| Moroccan | 228 (6.3) | 352 (7.4) |  |
| Cape Verdean | 111 (3.0) | 244 (5.2) |  |
| Surinamese-Hindustani | 79 (2.2) | 212 (4.5) |  |
| Surinamese-Creole | 115 (3.2) | 170 (3.6) |  |
| Antillean | 122 (3.4) | 173 (3.7) |  |
| Other non-western | 995 (27.3) | 0 (0.0) |  |
| Age (years) | 29.4 (5.6) | 29.9 (5.0) | <0.001 |
| Gestational age at iron blood sampling (weeks) | 13.7 (2.1) | 13.4 (2.0) | <0.001 |
| CRP (mg/L)^1^ | 4.3 (2.2, 7.9) | 4.6 (2.5, 8.2) | 0.002 |
| Monthly household income (Euros) n, (%) |  |  | <0.001 |
| <1200 € | 713 (25.7) | 668 (17.3) |  |
| 1200-2200 € | 710 (25.6) | 954 (24.7) |  |
| >2200 € | 1355 (48.8) | 2244 (58.0) |  |
| Educational level |  |  | <0.001 |
| Low | 494 (14.2) | 446 (9.7) |  |
| Intermediate | 1635 (47.0) | 2108 (46.0) |  |
| High | 1350 (38.8) | 2028 (44.3) |  |
| Parity, nulliparous (%) | 2151 (53.3) | 2710 (57.4) | <0.001 |
| Dietary iron intake (mg/day) | 10.5 (8.2, 12.9) | 11.1 (8.7, 13.5) | <0.001 |
| Iron supplement use, yes (%) | 951 (30.3) | 1201 (29.2) | 0.337 |
| Folic acid supplement use (yes), n (%) |  |  |  |
| No | 1010 (36.3) | 918 (24.3) | <0.001 |
| First 10 weeks use | 852 (30.6) | 1188 (31.4) |  |
| Periconceptional use | 919 (33.0) | 1673 (44.3) |  |
| BMI (kg/m) | 23.6 (4.4) | 23.7 (4.3) | 0.392 |
| Smoking during pregnancy, yes (%) | 883 (26.0) | 1228 (28.3) | 0.027 |
| Psychological distress, yes (%) | 367 (13.3) | 357 (9.2) | <0.001 |

In unique women with consent (N=9979, see Supplementary Figure 1), a comparison was made between non-participants and participants. Values are means (SD), ^1^medians (25-75th percentile) or valid percentages (absolute numbers) based on observed data. Pre-pregnancy body max index (BMI). C-reactive protein (CRP). P-value was calculated using Student’s T-test, Mann-Whitney U test and chi-square test.

**Supplementary Table S2.** Characteristics by iron status.

|  | Iron deficiency,  ferritin <15 µg/L  (n = 330) | Normal  ferritin between 15-150 µg/L  (n= 4089) | Iron overload  ferritin >150 µg/L  (n = 318) | Normal vs iron deficiency  p-value | Normal vs iron overload  p-value | Iron deficiency vs iron overload p-value |
| --- | --- | --- | --- | --- | --- | --- |
| Age (years) | 28.3 (5.4) | 29.9 (5.0) | 31.0 (3.8) | <0.001 | <0.001 | <0.001 |
| Gestational age at iron blood sampling (weeks) | 14.0 (2.1) | 13.4 (2.0) | 12.9 (1.9) | <0.001 | <0.001 | <0.001 |
| CRP (mg/L)^1^ | 5.1 (2.8, 9.0) | 4.5 (2.5, 8.2) | 4.6 (2.4, 8.1) | 0.086 | 0.778 | 0.320 |
| Monthly household income, n (%) |  |  |  | <0.001 | <0.001 | <0.001 |
| <1200 € | 81 (34.0) | 563 (16.8) | 24 (8.9) |  |  |  |
| 1200-2200 € | 77 (32.4) | 831 (24.8) | 46 (17.0) |  |  |  |
| >2200 € | 80 (33.6) | 1963 (58.5) | 201 (74.2) |  |  |  |
| Educational level, n (%) |  |  |  | <0.001 | <0.001 | <0.001 |
| Low | 68 (21.8) | 360 (9.1) | 18 (5.8) |  |  |  |
| Intermediate | 152 (48.7) | 1839 (46.4) | 117 (38.0) |  |  |  |
| High | 92 (29.5) | 1763 (44.5) | 173 (56.2) |  |  |  |
| Parity, nulliparous, n (%) | 129 (39.3) | 2323 (57.0) | 258 (81.4) | <0.001 | <0.001 | <0.001 |
| Dietary iron intake (mg/day) | 10.3 (8.3, 12.6) | 11.2 (8.8, 13.7) | 11.0 (8.7, 13.3) | 0.001 | 0.395 | 0.056 |
| Multivitamin use, yes (%) | 41 (14.5) | 1069 (30.0) | 91 (34.1) | <0.001 | 0.186 | <0.001 |
| Folic acid supplement use, n (%) |  |  |  | <0.001 | <0.001 | <0.001 |
| No use | 125 (47.0) | 765 (23.5) | 28 (11.0) |  |  |  |
| First 10 weeks use | 71 (26.7) | 1037 (31.8) | 80 (31.5) |  |  |  |
| Periconceptional use | 70 (26.3) | 1457 (44.7) | 146 (57.5) |  |  |  |
| BMI (kg/m) | 24.1 (4.6) | 23.6 (4.3) | 24.3 (4.6) | 0.087 | 0.012 | 0.540 |
| Smoking during pregnancy, yes (%) | 68 (22.8) | 1088 (29.0) | 72 (24.9) | 0.028 | 0.160 | 0.618 |
| Psychological distress, yes (%) | 48 (18.1) | 297 (8.9) | 12 (4.4) | <0.001 | 0.014 | <0.001 |

Values are means (SD), ^1^medians (25-75th percentile) or absolute numbers (valid percentages) based on observed data. Pre-pregnancy body max index (BMI). C-reactive protein (CRP). P-value was calculated using Student’s T-test, Mann-Whitney U test and chi-square test.**Supplementary Table 3** Ranking of factors relevant to ethnic differences in ferritin.


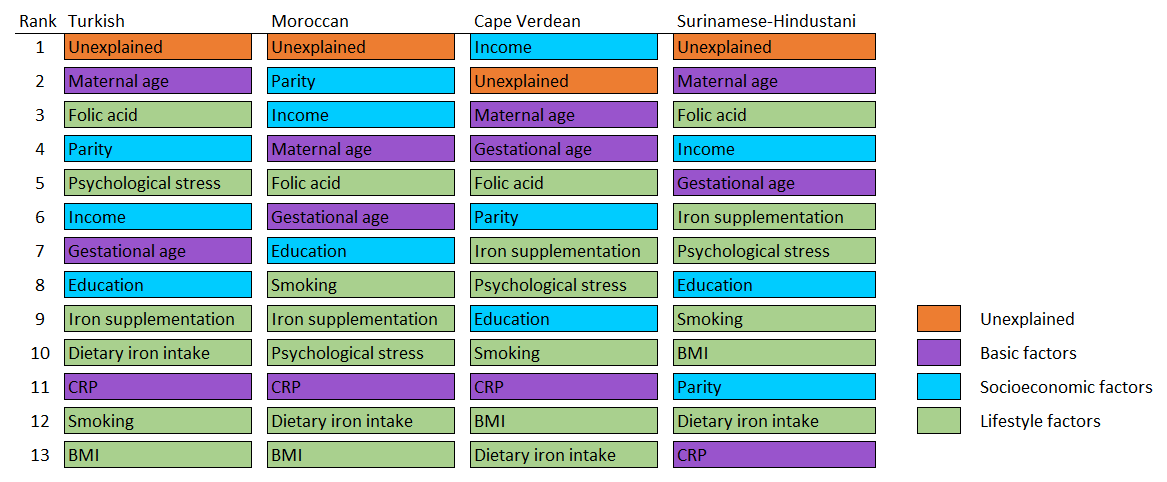


Contributing factors relevant to the differences in ferritin in consecutive order with the strongest on top, by ethnic group, based on the Oaxaca-Blinder analysis. BMI: Pre-pregnancy body mass index; CRP: C-reactive protein.

**Supplementary Table 4** **Secondary iron biomarkers and clinical outcomes in early pregnancy by ethnic background**

|  |  |  | **All** | **Dutch** | **Turkish** | **Moroccan** | **Cape Verdean** | **Surinamese-Hindustani** | **Surinamese-Creole** | **Antillean** |
| --- | --- | --- | --- | --- | --- | --- | --- | --- | --- | --- |
| **Iron biomarkers** | |  |  |  |  |  |  |  |  |  |
|  |  | N | 4737 | 3112 | 474 | 352 | 244 | 212 | 170 | 173 |
|  | **Iron** | (µmol/L) | 17.1 (6.6) | 17.9 (6.7) | **15.6 (6.1)**** | **16.2 (6.1)**** | **16.7 (6.2)*** | **13.8 (6.1)**** | **15.5 (5.5)**** | **15.3 (5.8)**** |
|  | **Trans ferritin** | (g/L) | 2.9 (0.5) | 2.8 (0.4) | **3.1 (0.5)**** | **2.9 (0.5)**** | **2.9 (0.5)**** | **3.2 (0.5)**** | 2.8 (0.4) | 2.8 (0.4) |
|  | **Transferrin saturation** | (%) | 24.5 (10.5) | 26.0 (10.6) | **20.7 (8.8)**** | **23.0 (10.2)**** | **23.5 (9.7)**** | **18.0 (9.1)**** | **22.7 (9.5)**** | **22.3 (9.8)**** |
|  |  |  |  |  |  |  |  |  |  |  |
|  |  | N | 4191 | 2711 | 448 | 316 | 219 | 192 | 154 | 151 |
|  | **Haemoglobin** | (g/dL) | 12.3 (0.9) | 12.5 (0.8) | **11.9 (0.9)**** | **12.2 (1.1)**** | **11.8 (1.0)**** | **12.0 (1.0)**** | **11.6 (0.9)**** | **11.9 (1.0)**** |
| **Clinical outcomes** | |  |  |  |  |  |  |  |  |  |
|  |  | N | 4191 | 2711 | 448 | 316 | 219 | 192 | 154 | 151 |
|  | **Anaemia^1^** | n (%) | 338 (8.1) | 99 (3.7) | **68 (15.2)**** | **33 (10.4)**** | **38 (17.4)**** | **28 (14.6)**** | **43 (27.9)**** | **29 (19.2)**** |
|  | **Iron deficiency anaemia** ^2^ | n (%) | 83 (2.0) | 10 (0.4) | **25 (5.6)**** | **14 (4.4)**** | **10 (4.6)**** | **16 (8.3)**** | 2 (1.3) | **6 (4.0)**** |

Values indicate median *25^th^, 75^th^ percentile), or number (percentage).^1^ defined as haemoglobin <11g/dL; ^2^ ferritin <15µg/L + haemoglobin <11g/dL. All groups where compared with the reference group (Dutch) using simple T-test, U-Mann-Whitney test or Chi-Square test as appropriate. Values that are significant different as compared to the Dutch reference group are indicated in bold (p<0.05), with * (p<0.01) or ** (p<0.001), based on T-test, Mann-Whitney U test and Chi-square test.

**Supplementary Table 5.** Ethnic differences in secondary outcomes transferrin saturation and haemoglobin during early pregnancy.

| Total  (n= 4737) | Dutch  (n= 3112) | Turkish  (n = 474) | Moroccan  (n = 352) | Cape Verdean  (n = 244) | Surinamese-Hindustani  (n = 212) | Surinamese-Creole  (n = 170) | Antillean  (n = 173) |
| --- | --- | --- | --- | --- | --- | --- | --- |
| **Transferrin saturation (z-score)** | | | | | | | |
| Basic model | Ref | **-0.5 (-0.6, -0.4)**** | **-0.2 (-0.3, -0.1)**** | **-0.2 (-0.3, -0.0)*** | **-0.7 (-0.8, -0.5)**** | **-0.3 (-0.4, -0.1)**** | **-0.3 (-0.4, -0.2)**** |
| SE model | Ref | **-0.4 (-0.5, -0.3)**** | -0.1 (-0.2, 0.0) | -0.1 (-0.3, 0.0) | **-0.7 (-0.8, -0.5)**** | **-0.2 (-0.4, -0.1)*** | **-0.3 (-0.4, -0.1)*** |
| LS model | Ref | **-0.4 (-0.5, -0.3)**** | -0.1 (-0.2, 0.0) | -0.1 (-0.3, 0.0) | **-0.7 (-0.8, -0.5)**** | **-0.2 (-0.4, -0.1)*** | **-0.2 (-0.4, -0.1)*** |
| Full model | Ref | **-0.4 (-0.5, -0.2)**** | 0.0 (-0.2, 0.1) | -0.1 (-0.2, 0.0) | **-0.6 (-0.8, -0.5)**** | **-0.2 (-0.3, -0.0)** | **-0.2 (-0.4, -0.1)*** |
| **Haemoglobin (z-score)** | | | | | | | |
| Basic model | Ref | **-0.6 (-0.7, -0.5)**** | **-0.3 (-0.4, -0.2)**** | **-0.7 (-0.8, -0.5)**** | **-0.5 (-0.7, -0.4)**** | **-0.9 (-1.1, -0.8)**** | **-0.7 (-0.8, -0.5)**** |
| SE model | Ref | **-0.6 (-0.7, -0.5)**** | **-0.3 (-0.4, -0.2)**** | **-0.6 (-0.8, -0.5)**** | **-0.5 (-0.7, -0.4)**** | **-0.9 (-1.1, -0.8)**** | **-0.6 (-0.8, -0.5)**** |
| LS model | Ref | **-0.6 (-0.7, -0.5)**** | **-0.4 (-0.5, -0.2)**** | **-0.7 (-0.8, -0.6)**** | **-0.5 (-0.7, -0.4)**** | **-1.0 (-1.2, -0.8)**** | **-0.7 (-0.9, -0.6)**** |
| Full model | Ref | **-0.6 (-0.7, -0.5)**** | **-0.3 (-0.5, -0.2)**** | **-0.7 (-0.8, -0.5)**** | **-0.5 (-0.7, -0.4)**** | **-1.0 (-1.1, -0.8)**** | **-0.7 (-0.8, -0.5)**** |

Effect estimates derived from linear regression models based on multiple imputed data, reported per ethnic group compared with the Dutch (reference) group. Values indicate beta coefficients (95% confidence intervals) reflecting mean difference; The basic model was adjusted for age, gestational age at iron blood sampling and C-reactive protein. The socioeconomic (SE) model was adjusted for the determinants in the basic model and for monthly household income, education and parity. The life style (LS) model was adjusted for the factors in the basic model and dietary iron intake, iron supplement use, folic acid supplement use, pre-pregnancy body mass index, smoking during pregnancy, and psychological distress. The full model included all these determinants named. Values that are significant are indicated in bold (p<0.05), with * (p<0.01) or ** (p<0.001).

**Supplementary Table 6** Ethnic differences in anaemia and iron deficiency anaemia during early pregnancy.

| Total  (n= 4737) | Dutch  (n= 3112)  OR | Turkish  (n = 474)  OR  (95% CI) | Moroccan  (n = 352)  OR  (95% CI) | Cape Verdean  (n = 244)  OR  (95% CI) | Surinamese-Hindustani  (n = 212)  OR  (95% CI) | Surinamese-Creole  (n = 170)  OR  (95% CI) | Antillean  (n = 173)  OR  (95% CI) |
| --- | --- | --- | --- | --- | --- | --- | --- |
| **Anaemia** |  |  |  |  |  |  |  |
| Basic model | 1.0 | **3.8 (2.7, 5.4)**** | **2.3 (1.5, 3.5)**** | **4.3 (2.9, 6.6)**** | **3.9 (2.4, 6.2)**** | **9.2 (6.1, 14.0)**** | **5.4 (3.3, 8v6)**** |
| SE model | 1.0 | **4.0 (2.7, 5.9)**** | **2.3 (1.4, 3.7)**** | **4.4 (2.8, 7.0)**** | **4.1 (2.5, 6.6)**** | **9.1 (5.8, 14.4)**** | **5.3 (3.2, 8.8)**** |
| LS model | 1.0 | **4.2 (2.8, 6.2)**** | **2.6 (1.6, 4.2)**** | **4.7 (3.0, 7.4)**** | **4.0 (2.5, 6.6)**** | **10.4 (6.6, 16.3)**** | **6.0 (3.6, 9.8)**** |
| Full model | 1.0 | **4.2 (2.8, 6.3)**** | **2.6 (1.6, 4.2)**** | **4.6 (2.9, 7.5)**** | **4.1 (2.5, 6.8)**** | **9.9 (6.2, 15.9)**** | **5.7 (3.4, 9.6)**** |
| **Iron deficiency anaemia** |  |  |  |  |  |  |  |
| Basic model | 1.0 | **10.1 (4.7, 21.9)**** | **7.5 (3.2, 17.5)**** | **7.8 (3.1, 19.6)**** | **17.0 (7.4, 38.9)**** | 2.5 (0.5, 11.6) | **6.9 (2.4, 19.9)**** |
| SE model | 1.0 | **7.2 (3.0, 17.1)**** | **5.0 (1.9, 12.9)*** | **5.6 (2.0, 15.2)*** | **13.7 (5.5, 33.7)**** | 1.7 (0.3, 8.4) | **4.7 (1.5, 14.7)*** |
| LS model | 1.0 | **6.0 (2.6, 14.1)**** | **4.3 (1.7, 11.1)*** | **4.7 (1.8, 12.5)*** | **11.3 (4.7, 27.1)**** | 1.7 (0.3, 8.1) | **4.8 (1.6, 14.6)*** |
| Full model | 1.0 | **5.3 (2.1, 13.3)**** | **3.7 (1.3, 10.1)** | **4.2 (1.5, 12.0)*** | **10.5 (4.1, 26.7)**** | 1.4 (0.3, 7.2) | **4.0 (1.2, 13.1)** |

Values indicate odds ratio (OR) with 95 confidence interval (95% CI), derived from logistic regression models based on multiple imputed data, reported per ethnic group compared with the Dutch (reference) group. Iron deficiency is defined as serum ferritin <15 µg/L, anaemia as haemoglobin <11 g/dL, iron deficiency anaemia as the combination of iron deficiency and anaemia. The basic model was adjusted for age, gestational age at iron blood sampling and C-reactive protein. The socioeconomic (SE) model was adjusted for the determinants in the basic model and for monthly household income, education and parity. The lifestyle (LS) model was adjusted for the factors in the basic model and dietary iron intake, iron supplement use, folic acid supplement use, pre-pregnancy body mass index, smoking during pregnancy, and psychological distress. The full model included all these determinants named. Values that are significant are indicated in bold (p<0.05), with * (p<0.01) or ** (p<0.001).

**Supplementary Figure 1.** Flow chart of the participants included in the analysis

**4737 included**

**for analysis**

9778 participating participations

in the Generation R study

n = 9778

8378 with data

on ethnic background

899 excluded,

802 due to no consent,

97 due to multiple repeated participation

1937 excluded,

due to no iron data available

8879 unique women

with consent

501 excluded,

due to no ethnic data available

6674 belonging to

large ethnic group

1704 excluded,

due to belonging to small (N < 40) ethnic subgroup

POPULATION

EXPOSURE

OUTCOME

**
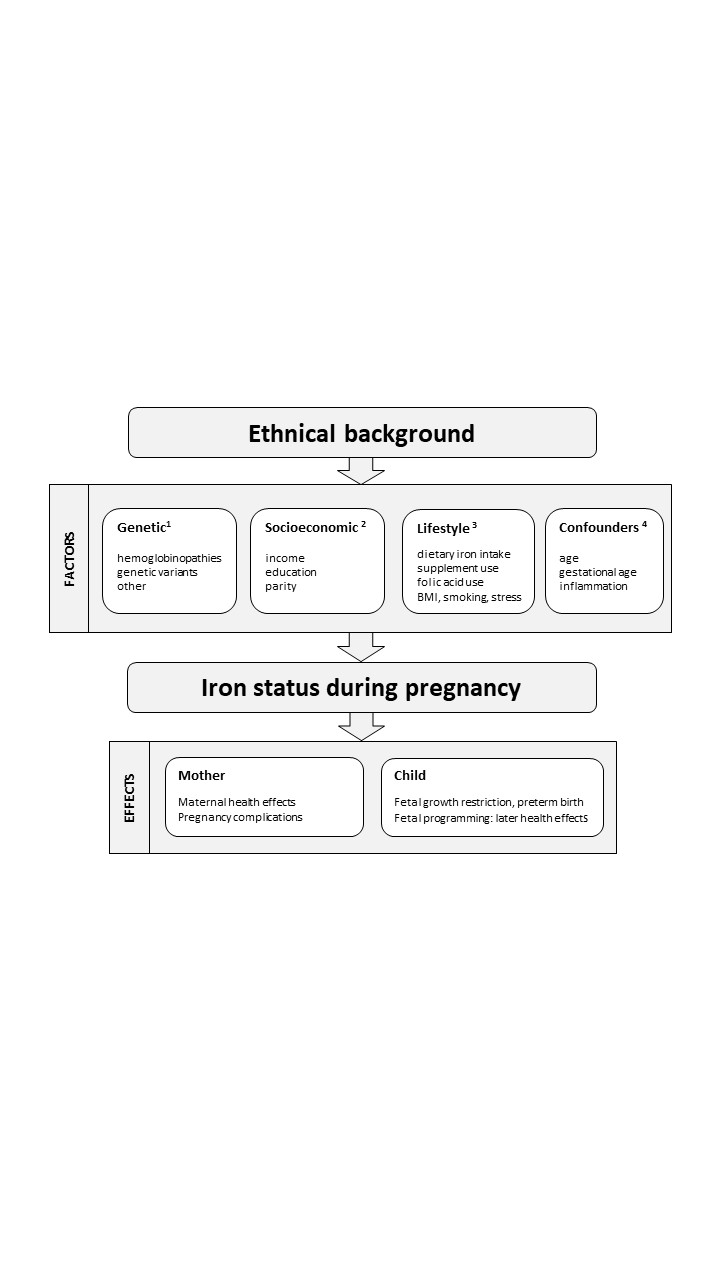
**

**Supplementary Figure 2.** Conceptual framework for ethnical differences in iron status during early pregnancy, that underlies the design of the multivariable models. ^1^ Genetic factors were not included in this study; ^2^ factors included in the socioeconomic and full model; ^3^ factors included in the lifestyle and full model; ^4^ potential confounders included in all models.

**Supplementary Figure 3.** Distribution of biomarkers by ethnic background.

**A**


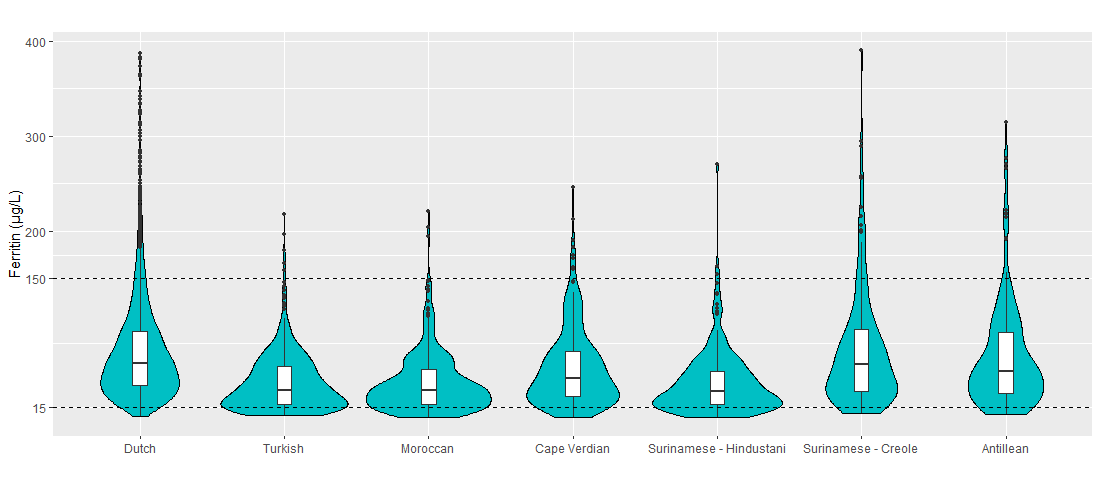


**B**


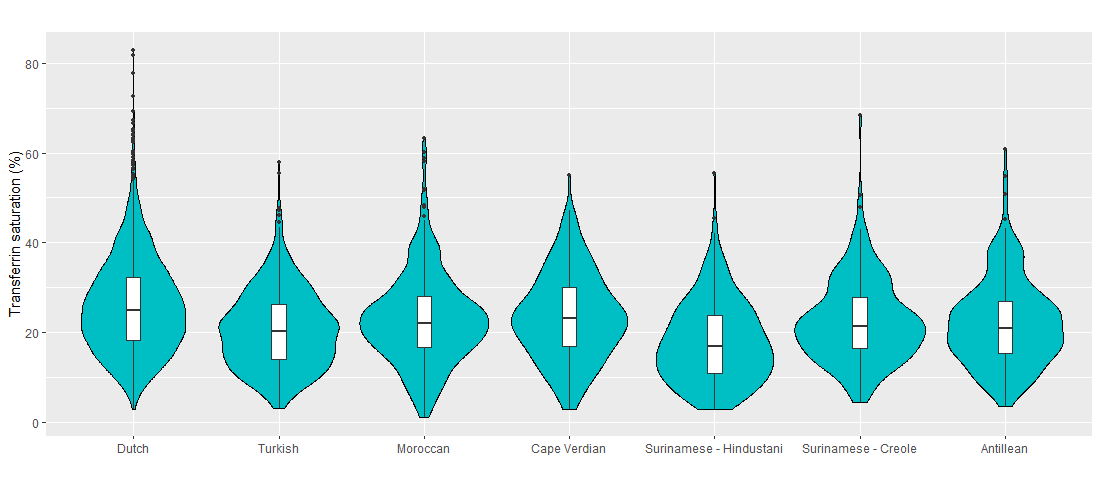


**C**


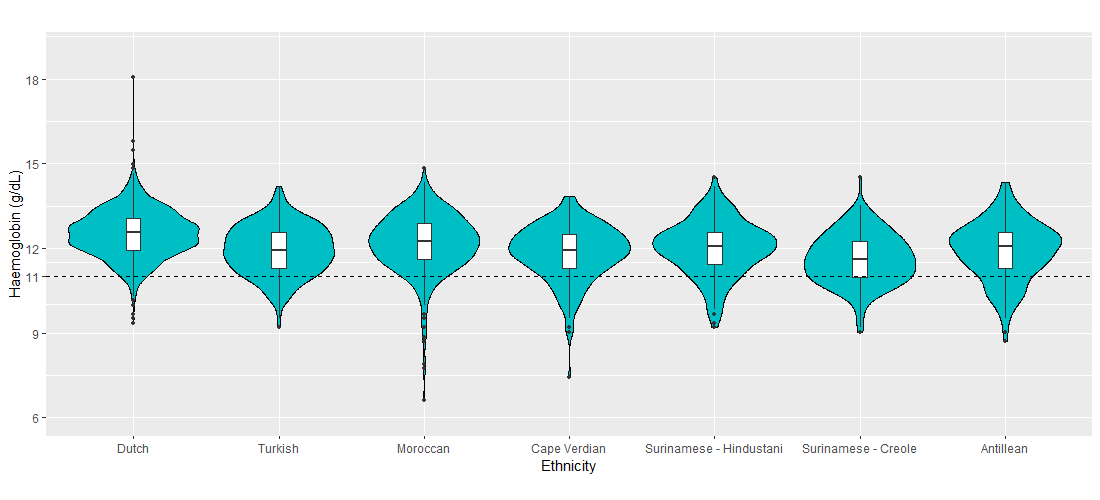


Violin plots of the distributions of ferritin concentrations (A), transferrin saturation (B), and haemoglobin (C) in early pregnancy by ethnic background. Box plots show first and third quartiles and the median, dots represent extreme observations. In A dashed lines represent cut-off values for iron deficiency (ferritin <15 µg/L), normal (ferritin 15-150 µg/L) and iron overload (ferritin >150 µg/L). In C dashed lines represent the cut-off for anaemia (<11 g/dL). Kruskal – Wallis and ANOVA test for mean differences was significant for serum ferritin and transferrin saturation respectively (p<0.001).
